# Secondary benefits of the families improving together (FIT) for weight loss trial on cognitive and social factors in African American adolescents

**DOI:** 10.1186/s12966-019-0806-5

**Published:** 2019-05-24

**Authors:** Allison M. Sweeney, Dawn K. Wilson, Haylee Loncar, Asia Brown

**Affiliations:** Department of Psychology, University of South Carolina, Columbia, SC 29208 USA

**Keywords:** Ripple effects, Family-based interventions, Health disparities

## Abstract

**Background:**

Although weight loss is an important primary outcome in obesity interventions, family-based interventions may have cascading ripple effects that extend to other aspects of health and well-being. Identifying these secondary benefits may be useful for understanding how to best engage underserved African American families in weight loss. The present research examines whether African American adolescents and parents perceive secondary benefits from participating in a family-based weight-loss intervention, including secondary health, social, or cognitive benefits.

**Methods:**

Qualitative data were obtained from families participating in the group-based intervention of the Families Improving Together (FIT) for Weight Loss trial. During the final week of the face-to-face motivational and family-based intervention program, families completed a guided open-ended group discussion about changes they experienced from participating (14 groups, *N* = 41 adolescents and 41 parents). Sessions were audiotaped, transcribed, and coded by independent pairs of raters using both inductive and deductive approaches. Guided by the multi-theoretical framework for the FIT trial, some themes were determined prior to coding using a deductive approach, including: (a) health outcomes (e.g., monitoring strategies for diet and physical activity), (b) social outcomes (e.g., involvement in family support, group support, autonomy support, family bonding, positive communication) and (c) cognitive outcomes (e.g., expression of self-confidence through self-efficacy, self-regulation, establishment of long-term goals). In addition to these pre-determined themes, the coding process included an inductive assessment, allowing for unexpected themes to surface as well around positive self-talk, relapse prevention, and monitoring strategies for different types of weight-related behaviors.

**Results:**

Across both adolescents and parents, the cognitive outcomes were the most frequently discussed outcomes, including self-regulation, monitoring strategies for diet, establishing long-term goals, and ultimate relapse prevention. Parents made a greater number of comments about the social outcomes, including family support, group support, self-efficacy, and family connectedness, whereas adolescents made a greater number of comments about positive family communication.

**Conclusions:**

The results provide preliminary support for the positive secondary effects of weight loss programs on improving both cognitive and social well-being in underserved African American adolescents.

**Trial registration:**

ClinicalTrials.gov # NCT01796067. https://clinicaltrials.gov/ct2/show/NCT01796067?term=NCT01796067&rank=1

The trial was registered on February 21, 2013 and the first participant was enrolled July 12, 2013.

## Background

Although reducing childhood obesity has been a national priority for several decades, in the last 15 years there has been no significant decline in obesity among children or adolescents [1]. Approximately 35% of children and adolescents in the U.S. are overweight or obese, with African American youth having significantly higher prevalence rates of overweight and all classes of obesity relative to White youth [1]. African American youth are also disproportionately impacted by medical complications related to being overweight, including impaired glucose tolerance, type 2 diabetes, and early signs of insulin resistance syndrome and cardiovascular risk [2, 3]. Furthermore, adolescent obesity can lay the foundation for a lifetime of health complications, as 70% of obese adolescents remain obese as adults [4], thereby increasing risk for elevated blood pressure, blood glucose, cholesterol, diabetes, and some cancers [5].

In line with the World Health Organization’s definition of health as including physical, mental, and social well-being [6], increasingly researchers are recognizing the importance of adopting a broad perspective on adolescent obesity that focuses not only on the absence of disease, but also integrates outcomes relating to physical, mental, and social well-being. Weight-related behaviors among youth, including unhealthy eating habits and low levels of physical activity, have been shown to cluster with other aspects of social and mental well-being, including higher levels of risk-taking and greater depressed mood [7–9]. Furthermore, evidence from randomized controlled trials and longitudinal studies have demonstrated that increases in physical activity also promote enhanced cognitive functioning in adolescents, including positive changes in memory, attention, executive functioning, planning, decision-making, and academic performance [10, 11]. There is also evidence that behavioral lifestyle interventions targeting improved diet and physical activity in adolescents result in improved self-regulation, including positive changes in self-efficacy, competency, and autonomy for diet and physical activity [12, 13]. Taken together, such studies suggest that intervening on adolescents’ weight-related behaviors (e.g., diet and physical activity) can result in cascading “ripple” effects across a range of outcomes relating to psychological and social well-being.

Wilson proposed that, to the extent that physical health clusters with psychological and social well-being, viewing behavioral interventions through the lens of ripple effects offers a valuable framework for enhancing the impact and cost-effectiveness of behavioral medicine [14]. However, to capitalize on this clustering, more research is needed to clarify the underlying cognitive and social mechanisms that give rise to cascading ripple effects across physical, psychological, and social outcomes. Given that support for ripple effects in behavioral interventions has been derived from quantitative analyses, relatively little is known about how adolescents perceive changes in weight-related behaviors as impacting their physical, psychological and social well-being. Developing a richer understanding of adolescents’ perceptions of the benefits of participating in a behavioral weight-loss intervention may serve to identify critical underlying cognitive and social mechanisms, which can be used to promote greater adolescent well-being in future behavioral interventions.

Importantly, previous studies examining ripple effects in adolescents have focused on ripple effects across different outcomes within a single adolescent [10–13], or ripple effects across peers within schools [15]. Relatively less is known about how changes in adolescent weight-related behaviors ripple across parent-adolescent dyads participating in family-based weight-loss interventions. Adolescent obesity has been associated with numerous family-related factors, including family cohesion, parenting style, family stress, and emotional climate [16, 17]. Lifestyle interventions that integrate behavioral strategies and involve parents are among the most successful approaches for treating adolescent obesity [18–20]. Parents play a central role in shaping healthy eating and physical activity at home, especially through parenting style and positive parenting practices (e.g., monitoring, positive communication) [21, 22]. By involving both the parent and adolescent, family-based interventions offer a rich framework for examining how changes in adolescents’ weight-related behaviors ripple across different aspects of the home environment and the parent-adolescent relationship. African American families face some of the highest rates of obesity but remain majorly underrepresented in lifestyle weight-loss interventions [23]. Thus, developing a deeper understanding of the ways in which African American adolescents and their parents benefit from participating in a family-based weight-loss program may help to advance understanding of the best strategies for engaging African American families in making sustainable lifestyle changes.

Expanding upon Wilson [14], the present study was undertaken to provide a comprehensive evaluation of African American families’ perceptions of the benefits arising from participating in a family-based weight-loss intervention. The present study used a qualitative approach to examine whether participating in the group-based intervention program from the Families Improving Together (FIT) for Weight Loss randomized controlled trial resulted in secondary benefits for both parents and adolescents in regards to their physical, psychological and social well-being. The FIT trial tests the efficacy of a motivational and family-based weight-loss intervention with cultural tailoring for reducing Body Mass Index (BMI) in overweight African American adolescents [24, 25]. The FIT group-based intervention was designed to improve social interactions between parents and adolescents by teaching positive parenting skills and incorporated cognitive mastery through behavioral skills training. By targeting both social and cognitive elements, the FIT trial is well-suited for detecting potential secondary effects on psychological, and social well-being.

The trial uses a multi-theoretical framework that integrates core constructs from Family Systems Theory [26] (positive parenting skills, communication skills), Self-Determination Theory [27] (autonomy support and motivation), and Social Cognitive Theory [28] (goal-setting, self-monitoring). These theories were used deductively as the guiding framework in the present study for evaluating whether adolescents and parents perceived positive changes in health-related factors (monitoring strategies for weight-related behaviors), family social dynamics (family support, group support, autonomy support, family connectedness, positive communication, mental health), and cognitive outcomes (e.g., self-efficacy, self-regulation, establishing long-term goals). Importantly, an inductive approach was also applied to allow for the identification of additional themes.

## Methods

### Study design

The FIT staff is a randomized controlled trial testing the efficacy of a culturally-tailored, motivational plus family weight loss (M + FWL) versus a comprehensive health education (CHE) program on decreasing z-BMI among overweight African American adolescents [24, 25]. The FIT trial uses a 4-group cohort design, with each cohort participating in a 16-week intervention with two phases: 1) an 8-week face-to-face group program; and 2) an 8-week online program. In both phases of the program, the parent-adolescent dyads are randomized to either the M + FWL or the CHE programs, resulting in a 2 (group treatment) X 2 (online treatment) design. The present study uses data collected during group discussions, which were conducted during week 8 of the face-to-face M + FWL program.

### Participants

Data from 41 parents and 41 adolescents who participated in the face-to-face M + FWL intervention were included in the present study. See Table 1 for demographic data.

**Table 1:** Participant Characteristics (*N* = 41 parents and 41 adolescents)

Variable	Value
Adolescent age *M* (*SD*)	12.78 (1.82)
Adolescent BMI percential	96.30%
Adolescent sex (% female)	63.40%
Parent age *M* (*SD*)	43.59 (8.13)
Parent BMI *M* (*SD*)	37.10 (9.71)
Parent sex (% female)	95.10%
Parent education *N* (%)	
9 to 11 years	3 (4.9%)
12 years	5 (12.2%)
Some college	17 (41.5%)
4-year college	6 (14.6%)
Professional degree	11 (26.8%)
Parent income *N* (%)	
<$10 K	5 (12.2%)
$10–24 K	12 (29.3%)
$25–39 K	12 (29.3%)
$40–54 K	5 (12.2%)
$55 K+	7 (17.0%)

### Recruitment

The FIT trial worked closely with local pediatric clinics, schools, and community partners for participant recruitment [29]. Culturally-tailored radio advertisements were also aired on radio stations, flyers were placed in community centers and libraries, and trained recruitment staff attended local community festivals and events. Interested families were screened for eligibility by phone and offered a welcome visit to learn more about the project. At the welcome visit, if eligibility criteria were met, parents and adolescents were given the opportunity to provide consent and enroll in the run-in phase. The University of South Carolina Institutional Review Board approved this study.

### Eligibility

Eligibility for families includes: 1) having an African American adolescent between the ages of 11–16 years old who is overweight or obese, defined as having a BMI ≥85th percentile for age and sex, 2) at least one parent or caregiver living in the household with the adolescent who was willing to participate, and 3) internet access. Adolescents were ineligible if they were already taking part in a weight-loss program, taking medication that could interfere with weight loss, or had a medical or psychiatric condition that would interfere with engaging in physical activity or dietary behaviors.

### Theoretical overview of the M + FWL intervention program

The intervention essential elements and curriculum incorporates elements from Family Systems Theory (FST) [26], Self-Determination Theory (SDT) [27], Social Cognitive Theory (SCT) [28], and cultural adaptations for African Americans (see Table 2). FST targets positive parenting skills (parenting style, monitoring, shared decision-making, communication) and highlights the importance of warm and supportive family interactions. Consistent with FST and research showing that an authoritative parenting style (high nurturance, moderate control and monitoring) promotes more positive health behaviors in adolescents, the M + FWL program focused on improving parenting skills around communication, autonomy support, and social support. SDT proposes that people are more likely to sustain health behavior changes when experiences are enjoyable and self-initiated. Consistent with SDT, the M + FWL intervention promoted positive parenting skills that allowed adolescents to have choice and input in their physical activity and dietary decision-making process. This process is important for increasing adolescent motivation for health behavior change. SCT also proposes that self-efficacy, or belief in one’s ability to succeed in a task, plays an important role in how people approach their long-term goals, such that those with higher self-efficacy tend to persist longer in their efforts and are better at coping with setbacks. Integrating behavioral strategies from SCT that support the development of self-efficacy, including goal-setting, action-planning, and self-monitoring, the M + FWL intervention supported parents and adolescents in developing weekly manageable goals for weight loss.

**Table 2:** Families Improving Together for Weight Loss Theoretical Essential Elements

Theory	Essential Element	Description
SCT	Self-Monitoring	Parents and adolescents monitor their caloric intake, energy expenditure, and weight, using a tool of their choice.
SCT	Goal Setting	Parents and adolescents set specific weight-loss goals together weekly, including intake, expenditure, and sedentary behavior goals.
SCT	Self-Regulation Skills	Parents and adolescents learn to identify personal barriers, substitute healthier alternatives, and provide positive reinforcements.
SDT & FST	Communication Skills	Parents and adolescents use positive communication strategies, including reflective listening, problem-solving, and shared decision-making, to discuss weight-loss behaviors.
FST	Parental Monitoring and Limit Setting	Parents monitor and track adolescent self-monitoring and goals, set limits with adolescents around weight loss behaviors, and monitor implementation of family rules and rewards for adhering to weight-loss behaviors.
SCT, SDT, & FST	Social Support	Adolescents use strategies for eliciting social support for weight-loss behaviors from parents. Parents provide adolescents with social support for weight-loss behaviors.
SDT	Autonomy Support	Adolescents have choices and are provided with opportunities to give input. Parents seek input from adolescents and negotiate rules and behavior changes together. Families engage in shared decision-making.
SCT	Self-Efficacy	Adolescents and parents have opportunities to practice and successfully master weight loss strategies.
SDT	Motivation	Families provide input and build confidence in changing adolescent weight-loss behaviors.
Cultural Tailoring	Adaptation to Cultural Issues	Families develop action plans for resolving cultural barriers to weight loss and parenting skill development as appropriate.

Additionally, the M + FWL intervention integrated several approaches for cultural targeting and tailoring, including integrating program materials and topics that are important in African American culture, including identifying foods with special meaning, discussion of emotional eating, and hairstyle during physical activity. These cultural topics were developed based on input from participants in Cohorts 1 and 2 [24]. For further information about how cultural tailoring was implemented in the M + FWL online program, please see Wilson et al. 2019 [30].

### Face-to-face M + FWL weekly sessions

The M + FWL intervention was delivered one night per week for eight weeks in a private office building. Two trained facilitators led each group, with group sessions lasting approximately 90 min. Facilitators underwent extensive training and certification, which was led by senior members of the research team, including the PI, a co-investigator with extensive clinical training, and/or the intervention coordinator. Trainings included didactic and hands-on training in behavioral skills (related to weight loss, positive parenting, family communication strategies), motivational interviewing skills, techniques for promoting a positive social environment, and cultural competency skills. Facilitators were provided a facilitator’s guide for each session that included session objectives, key content, activities, and prompts for each session.

Families received curriculum with hands-on activities that correspond with the facilitator guides. Topics covered during the program included self-monitoring strategies, positive communication, effective goal-setting, energy-nutrition balance, social support, reducing screening time, and addressing barriers for physical activity. The FIT trial focused on a series of weight-related behaviors including, improving energy balance, fruit and vegetable consumption, and physical activity, and reducing sedentary behavior, consumption of sugar-sweetened beverages, and fast food. In addition to participating in the group sessions, families spent 10–15 min in a one-on-one feedback session with the facilitator, during which parents and adolescents submitted self-monitoring logs for the facilitator to review, received feedback on their weekly progress, developed a new goal and action-plan for the week, and problem-solved barriers.

### M + FWL final group-based discussion

In the final week of the face-to-face M + FWL program**,** the facilitators reviewed skills and discussed relapse prevention and strategies for dealing with emotional eating. Additionally, guided by the facilitators, the final 45 min of this session was dedicated to “family testimonials,” during which families were invited to discuss the changes they have experienced from participating in the M + FWL program. Specific prompts included, 1) “How will you continue to make changes and maintain the changes you have already made?”; 2) "What was helpful and for you and your family?; and 3) “What are your new goals moving forward?” Facilitators used motivational interviewing strategies (e.g., open-ended questions, reflecting, summarizing) [31] to probe for additional details in order to evoke rich comments from parents and adolescents.

A total of 14 group sessions were included in the present analyses. Groups ranged in size from one to five families. On average, 60% of families were in attendance during the final week of the program.^1^ The family testimonials were audiotaped and transcribed and checked for accuracy by independent pairs of graduate assistants.

### Qualitative analysis

Guided by the essential elements derived from FST, SCT, and SDT, a preliminary coding book was developed to code the family testimonials for secondary benefits of the M + FWL program in parents and adolescents separately. Using a deductive approach [32], codes were developed prior to coding the data that corresponded to the essential elements of the FIT intervention. Specifically, drawing from FST and SCT, which both highlight the importance of monitoring, a code was developed to classify discussion of monitoring strategies. FST was used to guide the development of codes for positive parenting outcomes and family dynamics, including codes for the discussion of family support, group support, autonomy support, family connectedness and/or bonding, positive communication, and mental health benefits. Finally, SCT was used to develop codes around self-efficacy and behavioral skills, including codes for self-confidence through self-efficacy, self-confidence through self-regulation, and establishment of long-term goals. Additionally, the transcripts were reviewed using an inductive approach to look for other codes not captured through the preliminary theory-driven codes. This approach allowed for the identification of monitoring strategies related to a range of weight-related behaviors, including weight, physical activity, diet, and sedentary behavior. The inductive approach also led to the identification of two additional cognitive themes, positive self-talk and relapse prevention. Thus, both theory-driven (deductive) and data-driven (inductive) approaches were used to develop the final coding book [33].

Codes were used to separate participant responses into manageable “themes,” and themes were organized into three categories: health, social, and cognitive outcomes. The present study aimed to identify prominent secondary benefits arising from completing the M + FWL intervention. Thus, we report on all themes that were included in the coding manual, but we defined saturated themes as concepts discussed by at least eight adolescents or parents across a minimum of five group sessions.

### Training coders

A team of evaluators coded the family testimonials. Prior to coding, all evaluators underwent a training session with the PI who has extensive training in qualitative research.

The training included reviewing the coding manual, discussing coding rules, reviewing examples, and working individually to code themes on a sample transcript. A follow-up training session was conducted to discuss any questions the evaluators had prior to the administration of the fourteen transcripts. Initial percentage of agreement was 62% on independently coded transcripts, with coding pairs reaching 100% consensus on all final codes through discussion, for a similar approach see St George et al., 2017 [34].

## Results

Table 3 provides a summary of themes, including information on frequency (total number of times a theme was endorsed) and the number of sources (family testimonial sessions) in which each theme emerged. Overall, parents made a greater number of comments than adolescents. Valid themes were defined as concepts discussed by at least eight participants across a minimum of five group sessions, with saturation being reached for 12 out of the 15 themes for parents, and saturation being reached for 9 out of the 15 themes for adolescents. Note that families did not make any comments about mental health outcomes, so this theme will not be discussed further.

**Table 3:** Summary of themes for adolescents and parents

	Adolescents		Parents	
	Total Frequency	Total Sources	Total Frequency	Total Sources
Health Outcomes				
Monitoring Strategies for Weight	1	1	1	1
Monitoring Strategies for PA	9*	5*	10*	5*
Monitoring Strategies for Diet	23*	11*	52*	13*
Monitoring Strategies for Sedentary Behavior	2	2	1	1
Social Outcomes				
Family Support	8*	5*	23*	11*
Group Support	3	3	25*	7*
Autonomy Support	4	4	8*	6*
Family Connectedness/bonding	9*	6*	14*	7*
Positive Communication	11*	6*	9*	7*
Cognitive Outcomes				
Self-Efficacy	8*	5*	22*	13*
Self-Regulation	45*	13*	84*	13*
Positive Self-Talk	2	1	8*	5*
Establishing Long-Term Goals	12*	7*	20*	12*
Ultimate Relapse Prevention	15*	8*	45*	11*

*Denotes themes that reached saturation (discussed by at least 8 participants across at least 5 sessions). *PA* physical activity

### Themes related to health outcomes

#### Monitoring strategies for diet and physical activity

Both adolescents and parents made comments about monitoring strategies for weight-related behaviors. Specifically, monitoring strategies for diet were the most frequently discussed. For example, one parent described how she will monitor her calories and make adjustments as needed to meet her weight-loss goal, “*When I count the calories … I know my goal is 1500 if I want to lose weight. And if I go over that, then I know, ok, don’t look to lose because you’ve gone over that. So the next meal I’ll go under so that, you know, I still get my 1500 even though I went over at breakfast. I know at lunch time, I can’t eat as much. Or at dinner time, I can’t eat as much*.” Relatedly, in reference to monitoring physical activity one adolescent explained how she planned to start using a smartphone app to track her exercise, “*It’s this little thing on my phone, it’s called “Better Me” and it’ll tell you what exercise you can do and tracks it for you.”*

### Themes related to social outcomes

#### Family support

Both adolescents and parents discussed ways in which the intervention program helped to improve family support. Parents made several comments about changes they observed around mealtime preparation, such as “*For my family members to be more active in making meals and everything because it’s really been helping out a whole lot. Like we’ve been making a lot of healthy meals together.*” In reference to discussion of how to handle setbacks, one adolescent focused on the importance of emotional social support within her family, “*One way we can support each other is taking care of each other and making sure we get through it together*.”

#### Group support

Although adolescents made relatively few comments about group support, many parents commented on the valuable support they received from the group environment. For example, one parent noted that, “*It’s been some trying times, but you know, we always could come up in here every week. And y’all just turn all that around*.” Similarly, another parent shared, “*And I want to thank all the parents for letting me know that I’m not the only one going through this. With my adolescent and their eating habits, I get a little worried there, but thank you for sharing and letting me know and giving me some helpful hints*.”

#### Autonomy support

Adolescents made relatively few comments about the importance of autonomy support, but the importance of autonomy support was a frequent theme among parents. For example, one parent noted that in order to achieve their long-term health goals, she recognized the importance of “*Including the adolescent in the decision making process.*” Relatedly, another parent commented about autonomy supportive strategies she learned for promoting healthier dietary choices in her adolescent, “*We talked about* [what] *we as parents let the adolescent take--be more involved with like, choosing the vegetable, when we go to the grocery store, suggesting a dish*.”

#### Family connectedness/bonding

Both parents and adolescents discussed ways in which the intervention program has improved their family connectedness and/or increased family bonding. One parent commented that, “*I’m going to continue to connect with my son and daughter, communicate with them, and let them know the importance of eating right*.” When discussing future goals, one adolescent commented that she wanted to make family connection a central goal by “*Spending time and asking questions and communicating*.” Another adolescent discussed how cooking as a family has helped her to feel closer to her family, “*We started cooking as a family. It makes a big difference*.”

#### Positive communication

Positive communication was another frequently discussed theme across both parents and adolescents. Adolescents most frequently noted this as an important change in the family interactions. One parent discussed some of the positive communication skills she learned through the intervention program, “*You know how we talked about communication, and listening, and reflecting back to make sure you heard what the other person said … giving eye contact to let the person know that you are paying attention to them, that you are listening*.” Furthermore, one of the adolescents described how communication with her mom has become more positive, “*Me and [my mom] actually had a good, decent conversation. And just like talking about stuff and like usual life problems, and no one got upset and no one got their voice raised at. It was cool*.”

### Themes related to cognitive outcomes

#### Self-efficacy

Across both parents and adolescents, there was frequent discussion of confidence in one’s ability to initiate or maintain positive weight-related behaviors. For example, one parent explained, “*We won’t look at our past mistakes as unobtainable but set goals each week that are obtainable and go forward with confidence that it will be achievable*.” One adolescent commented, in reference to peers judging her for making healthier dietary choices, that she was confident she could make healthy choices anyway, “*I don’t need a slice of pizza. If they’re going to look at me, then they are just going to have to look at me*.”

#### Self-regulation

Both parents and adolescents frequently noted discussion of their ability to make progress towards a healthier lifestyle, including setting goals, developing action-plans, appraising progress, and problem-solving as needed. For example, one parent described how she will keep working toward her weight-loss goal, “*I’m going to keep tracking. And just keep track of what I’m eating. And to just eat smaller portions. And make sure that I don’t skip meals*.” Similarly, one adolescent described positive actions she will continue to take moving forward, “*Count your calories, start watching what you eat, because it’s not like it’s a holiday every day. Don’t start making a bad habit and continue doing it every day.*”

#### Positive self-talk

Adolescents engaged in relatively little positive self-talk. However, parents made several comments in which they upheld a positive self-image and expressed self-encouragement for meeting their long-term weight-loss goals. For example, one parent described the importance of being patient with her weight loss progress, “*I have to be mindful that I didn’t put it on in 13 weeks, so I won’t be able to take it off in 13 weeks. So it will take some time to get it under control*.” Another parent expressed self-encouragement for continuing to make positive changes, “*I know doing the way I’m doing now, I will definitely get there. I’ve lost 7 pounds, and the medicine when I went back to the doctor, my blood pressure was excellent and my blood sugar was excellent. I know if I keep going, I’ll be off the medicine*.”

#### Establishing long-term goals

Parents and adolescents described creating reasonable goals to work towards and striving towards a permanent healthy lifestyle. One parent described strategies she learned through the intervention program that would support her long-term weight loss goal, “*For me, it was portion size. I learned the importance of watching your intake as well as keeping up with your outtake. I learned how important that was. And my goal is to keep it up so I can drop 50 pounds*.” One adolescent described how she would continue to use monitoring strategies to work toward her weight-loss goal, “*I plan to continue to track at least one day a week and weigh myself at least once a week, just like in our program. And my new goals are going to be to continue to lose weight*.”

#### Ultimate relapse prevention

Parents and adolescents frequently described how they would persevere during tough times, makeup for a missed workout, and plan around temptations. For example, one parent described how she would deal with temptations around the holidays, “*And when you know these holidays and birthdays are coming up … cut back on certain stuff ahead of time so that you’re giving your body the chance to be able to handle a little extra*.” When describing how she plans to stick with her weight-loss goal during recovery for an upcoming surgery, one adolescent explained, “*Ok, a tough situation for me will be going through foot surgery … As a teen, I would make a menu to stay within the calorie goals for that day.”*

## Discussion

The present study used a qualitative approach to examine whether parents and adolescents perceived secondary health, social, and cognitive benefits associated with participating in the group-based weight loss intervention program during the FIT trial. Regarding health outcomes, both parents and adolescents made frequent comments about the benefits of monitoring strategies for diet. In reference to social outcomes, parents made frequent comments about the importance of family support, group support, while adolescents benefited more from the positive communication with parents. Both parents and adolescents indicated they received benefits related to several cognitive outcomes, including self-regulation and relapse prevention. Taken together, these results demonstrate that adolescents and parents perceived a wide range of secondary health, social, and cognitive benefits that extended beyond weight-related health behavior change that was the target of the FIT intervention trial. The findings from this paper are one of the first to examine secondary ripple effects across African American parent-adolescent dyads participating in a family-based weight-loss intervention.

A key finding in the current study was the importance of social support from both group participants as well as from family members for the parents/caregivers involved in the FIT intervention trial. This finding is consistent with a growing literature that shows that social support is a highly valued construct among African American women in their weight loss efforts. For example, Barnes and colleagues [35] conducted focus groups with African American women to examine what factors were important in maintaining their weight loss. The results indicated that positive social support from others was critical for weight maintenance and that having strategies in place to prevent relapse was necessary for sustained weight loss. Relatedly, systematic literature reviews have revealed that general lack of social support and lack of someone to be physically active with are two of the most frequently cited interpersonal barriers among African American women [36].

Another key finding was the importance of positive communication with parents for adolescents in the FIT trial. In previous work by our group [37] we examined whether parent communication moderated the impact of a family-based intervention on sedentary behavior in African American adolescents. The results indicated that families receiving the intervention and who had reported higher rates of positive communication showed lower adolescent sedentary behavior than those with less positive communication or those in the comparison condition. Although no significant effects were found for the model with weight status as the outcome the weight range included those of normal weight, thereby restricting the range for significant weight loss [37]. Consistent with our previous work, the results from the present study indicate that communication between parent and adolescents may be an important factor to consider in the development of future health promotion programs for African American adolescents.

Other notable findings in this study included the high reporting of cognitive advances learned in the program for both parents and adolescents which included increasing their skills for monitoring, self-regulation, and relapse preventions. These findings are consistent with other large national trials that included racial minorities but which did not focus solely on sub-analyses within African American populations [38–40]. For example, Anderson and colleagues [38] examined social cognitive determinants of physical activity in churches that included 21% African American adults as part of a health promotion trial. Results indicated age, race, social support, self-efficacy, and self-regulation significantly contributed to participants’ physical activity levels. Of these, self-regulation exerted the strongest effect on physical activity. Social support also influenced physical activity as a direct precursor to self-efficacy and self-regulation. Elfhag and Rossner [39] reported in a literature review that successful weight maintenance was associated with self-monitoring of food intake and physical activity consistent with the Look AHEAD trial results [40]. Importantly, however, the current study is the first to evaluate the cognitive secondary benefits of a weight loss program in solely African American parents and their adolescents through qualitative methods.

There are several limitations to the present study that should be noted. First, adolescents made fewer comments than parents. Although the present trial implemented several novel approaches aimed at maximizing family engagement (e.g., cultural targeting and targeting positive parenting practices), future family-based interventions will need to continue to consider the best approaches for increasing engagement among adolescents. Another limitation is that these findings cannot be generalized beyond African Americans in the Southeastern United States. Although this study is somewhat limited in that only the intervention arm of the trial provided testimonials for qualitative analysis, it is one of the few trials to actively code these secondary gains through qualitative methods. The present results speak to the rich information that can be gained from qualitative analyses. Future family-based interventions should consider adopting a mixed-methods approach in order to gain further understanding of how the magnitude of behavior change (e.g., diet, exercise, weight loss) corresponds to perceptions of changes in cognitive, social, and mental well-being. Additionally, investigators are encouraged to use a more comprehensive approach to measuring physical, psychological, and social outcomes in future family-based health promotion trials.

## Conclusions

The results provide preliminary support for the positive secondary effects of weight loss programs on improving both cognitive and social well-being in underserved African American adolescents and their parents. Increasingly, researchers are recognizing the value of adopting a multi-behavioral model of behavioral medicine that encompasses physical, social, and mental health outcomes [14]. The present findings pinpoint several critical targets, including family support, family bonding, and the development of self-regulatory skills, which may be important mechanisms for engaging African American families in future weight-loss interventions. An important next step for understanding ripple effects will be to systematically test how underlying cognitive and social mechanisms, such as self-regulation and family cohesion, impact a broad range of outcomes related to well-being and quality of life. Furthermore, continuing to build systematic evidence that the positive effects of behavioral interventions extend beyond the absence of disease may be an effective approach for demonstrating to policy makers that behavioral medicine innterventions have a high return on investment.

## Abbreviations

BMI: Body mass index

## Footnotes

1 In general, other family members were not present during the family testimonials. There was one case in which an additional sibling sat in on the group discussion, but her comments were not coded as part of the discussion.
